# Antibacterial properties of lactoferrin: A bibliometric analysis from 2000 to early 2022

**DOI:** 10.3389/fmicb.2022.947102

**Published:** 2022-08-17

**Authors:** Yunling Xu, Yuji Wang, Jiaolong He, Wanping Zhu

**Affiliations:** ^1^Department of Basic Medical, Zhejiang Academy of Traditional Chinese Medicine, Hangzhou, China; ^2^Department of Intensive Care, First Affiliated Hospital of Jishou University, Jishou, China

**Keywords:** lactoferrin, antibacterial, bibliometrics, hotspots, research status, COVID-19

## Abstract

**Background:**

Here, a bibliometric and knowledge map analysis are used to analyze the research hot spots and development trends regarding the antibacterial effect of lactoferrin (LF). By looking for research hot spots and new topics, we provide new clues and research directions for future research.

**Methods:**

Articles and reviews regarding the antibacterial effect of LF were retrieved and from the Web of Science Core Collection (WoSCC) on 25 June 2022. CiteSpace and VOSviewer were used to conduct the bibliometric and knowledge map analysis.

**Results:**

In total, 8,292 authors at 2,151 institutions from 86 countries published 1,923 articles in 770 academic journals. The United States was the leader regarding research on the antibacterial effects of LF, while the Netherlands was a pioneer in conducting research in this field. The University of California system contributed the most publications. Bolscher JGM published most articles, while Wayne Bellamy had most cocitations. However, there was insufficient cooperation among the various institutions and authors. *BioMetals* published most LF-antibacterial activity-related articles, whereas *Infection and Immunity* was most commonly cocited journal. The most influential research hot spots about the antibacterial effect of LF focused on antimicrobial peptides, casein, human milk, expression, and *Escherichia coli*-related research. The latest hot spots and research frontier included COVID-19, antibiofilm activity, and immune defense.

**Conclusions:**

LF is a multifunctional protein with a broad spectrum of antimicrobial activities. The related field of antibacterial properties of LF will remain a research hot spot in future.

## Introduction

Lactoferrin (lactotransferrin, LF) is an ~80-kDa non-hem iron-binding glycoprotein of the transferrin family (González-Chávez et al., 2009). LF is widely present in many mammalian secretions, including mammalian milk, saliva, tears, bronchial and intestinal secretions, and secondary granules of neutrophils (Katunuma et al., 2003). Known as a natural antibiotic, it is an important component that bridges the innate and adaptive immune systems of mammals and plays roles in protecting human cells at all stages of life (Spadaro et al., 2008; Kowalczyk et al., 2022). LF is endowed with extensive biological properties, including antibacterial, antiviral, anti-inflammatory, antioxidant, anticarcinogenic, immunomodulatory, and enzymatic activities (García-Montoya et al., 2012; da et al., 2021). LF derived from natural bovine milk has been widely applied in fortifying food products (Kim et al., 2010; Carnicelli et al., 2021). The first discovered and the most well-known characteristic was its antibacterial property (Arnold et al., 1977). It has been shown to have broad-spectrum antibacterial inhibitory activity against Gram-positive bacteria, Gram-negative bacteria, and fungi, effectively inhibiting the growth of *Escherichia coli, Salmonella typhi, Streptococcus, Legionella pneumophila*, and *Staphylococcus aureus*. Clinically, oral treatment with LF inhibits the growth of gastrointestinal anaerobes but stimulates the proliferation of probiotic bacteria (de Sá Almeida et al., 2020). Consequently, it is significant to quantitatively analyze the *status quo*, focus areas, and future prospects related to LF antibacterial function research.

Bibliometrics is a method for assessing and monitoring the progress of specific disciplines via statistical analysis of published data (Li et al., 2020). Bibliometric analysis can be used to determine the outputs and citations of countries, institutions, and authors and the keyword frequency of research hot spots and frontiers in particular fields (Luo et al., 2021). Using bibliometric techniques, this study sought to provide a 22-year longitudinal view (2020–2021) of the evolution of the scientific literature on the antibacterial activities of LF. The published literature was primarily analyzed using the following criteria: publication year, country, affiliation, journal, author, keyword, citation, and H-index. Finally, the bibliometric analysis results were combined with a traditional review conducted under the guidance of bibliometrics to demonstrate the evolution of the research on the antibacterial activities of LF. This is the first attempt to conduct a statistical analysis of the literature on the antibacterial activities of LF. In addition, it will help researchers determine future research trends and give them useful tools for making decisions.

## Materials and methods

### Data collection

The Science Citation Index (SCI) Expanded (SCIE) of the Web of Science Core Collection (WoSCC) database was used to obtain bibliographic data. To avoid deviations caused by daily database renewal, all documents published between 2000 and early 2022 were retrieved and downloaded from the SCIE of WoSCC database on 25 June 2022. Search terms were as follows: TS = (lactoferrin OR lactoferrins OR lactoferrine OR lactoferricins OR lactotransferrin OR LF OR Lf OR LTF) AND TS = (antibacterial OR anti-bacterial OR antimicrobial OR anti-microbial OR antimycotic OR anti-mycotic OR antifungal OR anti-fungal). The language was limited to English, and only research articles and reviews were retrieved. For this, two investigators (YLX and JLH) independently conducted the primary data search and discussed any discrepancies. The final agreement reached a value of 0.90, indicating a substantial agreement (Landis and Koch, 1977). The data were saved and stored in a download_txt format. This study excluded proceedings papers, meeting abstracts, editorial materials, book chapters, corrections, early access, and letters.

### Data analysis

Microsoft Office Excel 2010 was used to manage data and analyze annual publications. The number of publications (NP) was used to measure productivity, and the number of citations without self-citations (NC) was used to represent the impact. The H-index was used to evaluate the academic contribution and predict future scientific achievements. For visual analysis, all valid data collected from the SCIE of WoSCC database were imported into VOSviewer (version 1.6.16) and CiteSpace (version 5.8.R1). The collaborative networks between countries, institutions, journals, and authors and cocitation of keyword clusters were visually analyzed using VOSviewer (van Eck and Waltman, 2010). CiteSpace was used to analyze the research progress; investigate the research status, hot spots, and trend distribution maps over time; and determine the field development trend (Chen, 2004; Ma et al., 2021). The research status and development trajectory of this field can be fully understood by examining keyword frequency, centrality intensity, and prominence. A coword network was constructed based on the keyword co-occurrence, with each node representing a keyword. When two keywords appear in the same article, they form a co-occurrence relationship and are represented in the network as a single edge. A large mean value indicates that the node has significant representation in a particular subject field at a specific time. The degree of emergence suggests how much the collinear frequency of nodes and the number of co-occurrence increase over time. The greater the degree of emergence, the more the evidence that the node was a research hot spot during a given period.

Burst detection, an algorithm developed by Kleinberg (2003), is an effective analytic tool for capturing rapid increases in the popularity of references or keywords over a specified time period. This function can quickly identify concepts or topics that are actively discussed during a specified period. The blue lines represent the time period in this graph, and the red lines represent the period when the reference burst occurred.

## Results

### Publication overview

**Figure 1:**
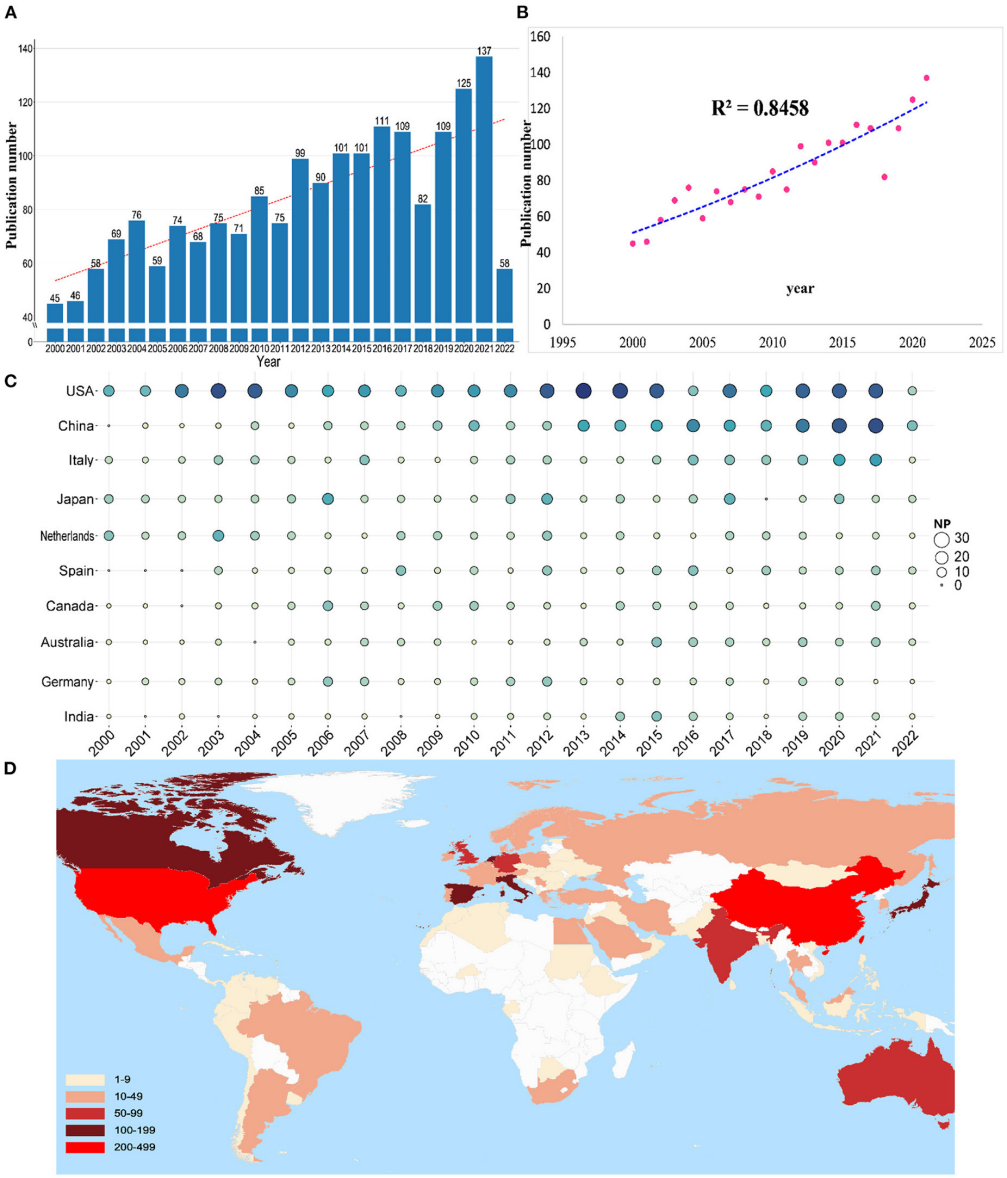
**(A)** Number of publications by year over the past 22 years. **(B)** Curve fitting of the total annual growth trend of publications (*R*^2^ = 0.8458). **(C)** Annual output trend of the top 10 productive countries (the size and colors of the circle represent the NP). **(D)** Publication counts distribution per country/region.

The total NP over a given period can objectively and quantitatively reflect the overall development trend of a field. A total of 1,923 publications were chosen based on the defined search terms, including 1,638 original articles (85.18%) and 285 reviews (14.82%). The annual NP is shown in Figure 1A. Despite some fluctuations, the NP increased from 45 in 2000 to 134 in 2021. Over the last 22 years, the growth rate has been relatively stable (the year 2022 was not included in the analysis as it is incomplete). Figure 1B depicts a polynomial fitting curve for the publications' total annual growth trend. The annual NP trended upward and was positively associated with the year of publication (R^2^ = 0.8458). Overall, these findings indicate that research on the antibacterial effects of LF has attracted increasing attention. Figure 1C illustrates yearly NP of the topmost influential countries. The circle size and color correspond to the NP values of the articles. Research in the United States on the antibacterial effects of LF is at the core of global studies, and research in China has been more prolific in recent decades. Figure 1D depicts the global distribution of publications by countries/regions, and results indicate uneven global distribution of the LF antibacterial research.

The publications were produced by 86 countries/regions, and 73 countries/regions had more than two publications. The 10 topmost influential countries are listed in Table 1, along with their NP, NC, H-index, and average citations per item (AC). The 10 topmost countries published 83.78% (1,611/1,923) of the publications. The United States was the leading country in terms of NP (23.50%, 452/1,923), followed by China (12.74%, 245/1,923), and Italy (8.27%, 159/1,923). The top three countries with the highest NC were the United States (19,824), Canada (7,609), and Italy (4,732). The top three countries with the highest H-index were the United States (75), Canada (74), and the Netherlands (44).

**Table 1:** Topmost 10 productive country in the field of LF antibacterial research.

**Rank**	**Country**	NP*	NC*	**H-index**	AC*
1	USA	452	19,824	75	45.38
2	China	245	4,381	37	18.77
3	Italy	159	4,732	39	31.34
4	Japan	150	3,574	34	25.30
5	Netherlands	127	5,610	44	48.17
6	Spain	108	2,866	31	28.04
7	Canada	105	7,609	74	38.00
8	Australia	97	2,989	34	31.76
9	Germany	96	4,018	33	42.24
10	India	72	2,154	22	30.11

^*^NP, total number of publications; NC, total number of citations; AC, average citations per item.

**Figure 2:**
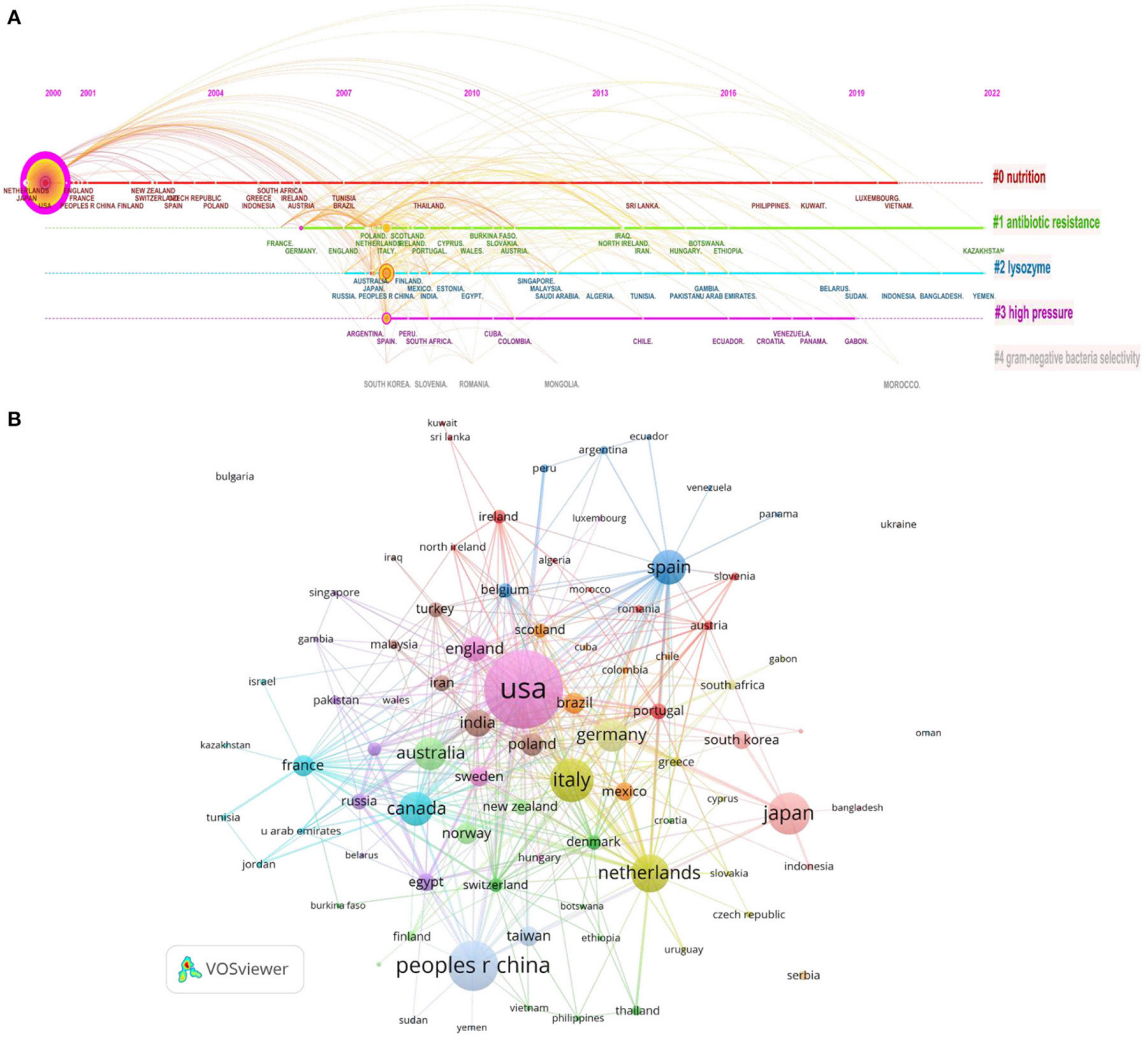
**(A)** Timeline view of country coauthorship cluster analysis in the LF antibacterial activity research. **(B)** Country coauthorship network visualization map.

CiteSpace is used to cluster countries and identify research hot spots, and the “log-likelihood ratio” is selected as the algorithm for extracting group inscriptions. As shown in Figure 2A, the node size and color on the horizontal line represent the NP and year range of occurrence, respectively. Purple outlines indicate those articles with significant betweenness centrality, and red nodes indicate frequently cited references (Kaur et al., 2017). In addition, the uppermost line shows the timeline for different fields, and the number longitudinal lines describe the distinct categories of LF antibacterial activity research, which are arranged vertically in descending order of the cluster size; the smaller the number of clusters, the more countries are included, and each cluster is composed of multiple closely related words. Specifically, early research on LF antibacterial activity was conducted on nutrition. Subsequently, studies were extended to antibiotic resistance, lysozyme, high pressure, and Gram-negative bacterial selectivity. As shown by the connecting arcs in Figure 2A, categories #0: nutrition interacts positively, but cross-international collaborative research on the Gram-negative bacterial selectivity remaining categories is inactive.

VOSviewer was used to analyze the coauthorship among different countries and produce visualization maps of international cooperation (articles coauthored by authors in more than one country are counted repeatedly by VOSviewer). Closely related terms are grouped into the same cluster with the same color. The more publications a country has produced, the larger the size of its circle will be; the larger the scale of the cooperation is, the thicker the connecting line will be. Figure 2B illustrates the country coauthorship network. The United States, the most productive country, showed the most cooperation with other countries, followed by the Netherlands, Germany, Italy, and Spain. These countries had close collaborations with other countries, which demonstrated that these countries emphasized research collaborations.

## Institutions

More than 2,151 institutions have made contributions to this field, with 135 producing more than six articles. Table 2 summarizes the top 10 most influential institutions with the highest NP related to the antibacterial effects of LF. The League of European Research Universities had the highest NP (122), followed by the University of California system (79) and Vrije Universiteit Amsterdam (53). The most significant AC scores were from the University of Calgary (109.60), followed by the University of California, Davis (66.65), and the University of California system (61.77).

**Table 2:** Topmost 10 productive institutions in the field of LF antibacterial research.

**Rank**	**Institution**	**Country**	NP*	NC*	AC*	**H-index**
1	League of European Research Universities	Netherlands	122	4,014	39	35.23
2	University of California System	United States	79	4,796	41	61.77
3	Vrije Universiteit Amsterdam	Netherlands	53	2,056	27	44.19
4	University of California Davis	United States	49	2,947	31	66.65
5	Academic Center for Dentistry Amsterdam	Netherlands	44	1,893	24	48.41
6	University of Amsterdam	Netherlands	36	828	19	27.03
7	Egyptian Knowledge Bank	Egypt	33	607	15	19.24
8	The Arctic University of Norway	Norway	32	1,816	26	59.25
9	Sapienza University Rome	Italy	31	1,089	19	36.42
10	University of Calgary	Canada	30	3,205	20	109.60

^*^NP, total number of publications; NC, total number of citations; AC, average citations per item.

**Figure 3:**
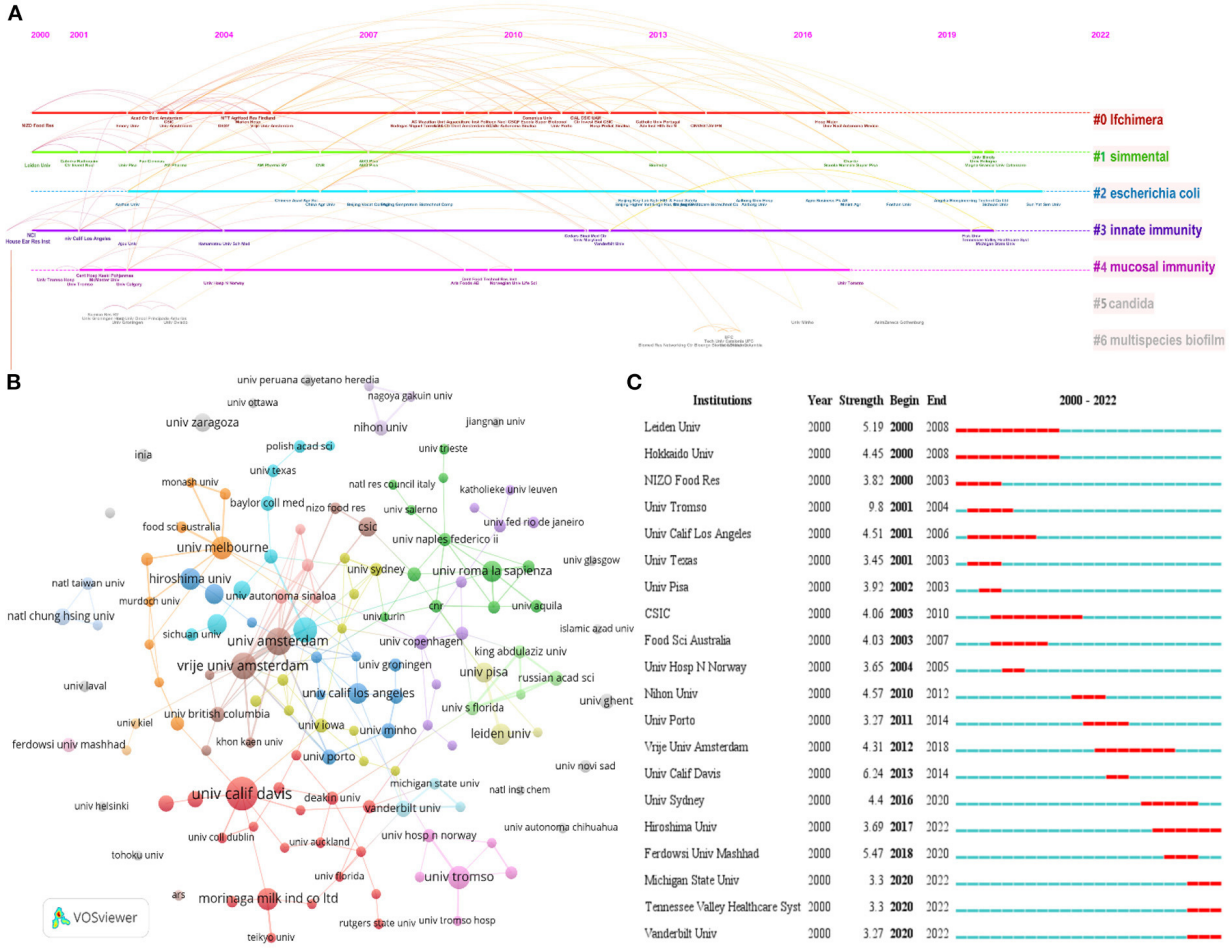
**(A)** Timeline view of institutions coauthorship cluster analysis in LF antibacterial research. **(B)** Institutions' coauthorship network visualization map. **(C)** Top 20 institutions' coauthorship with strong occurrence bursts.

The institutional visual cluster analysis is shown in Figure 3A, with the cluster labels on the right: “#0: LF chimera”, “#1: Simmental”, “#2: *Escherichia coli*”, “#3: innate immunity”, “#4: mucosal immunity”, “#5: Candida”, and “#6: multispecies biofilm.” The smaller the number of clusters is, the more institutions are included, and each cluster is composed of multiple closely related words. Figure 3B depicts the institutional coauthorship network. The University of Amsterdam had the highest number of collaborations, followed by Vrije Universiteit Amsterdam and the Autonomous University of Sinaloa. The present study applied the burst detection algorithm to extract active organizations for LF antibacterial research. The blue lines represent the time period in this graph, and the red lines represent the period when the reference burst occurred. Figure 3C illustrates the top 20 institutions with the strongest occurrence burst. In the recent past 3 years, the institutions with the strongest burst values were the Michigan State University, Tennessee Valley Healthcare System, and Vanderbilt University. Overall, the institutions were universities, and only a few were hospitals or enterprises.

### Authors and cocited authors

Over 8,396 authors have authored publications in this field. Among them, 138 authors contributed at least five articles to this collection. Table 3 summarizes the top 10 most active and productive authors. Bolscher JGM (University of Amsterdam) was the most prolific author (NP = 37, NC = 1,131) with the highest H-index (20), followed by Nazmi K (University of Amsterdam) and Nibbering PH (Leiden University). In addition, Vogel HJ (University of Calgary) had the highest AC (170.50).

**Table 3:** Top 10 active authors in the field of LF antibacterial research.

**Rank**	**Author**	**Affiliation**	**Country**	NP*	NC*	AC*	**H-index**
1	Bolscher JGM	Vrije Universiteit Amsterdam	Netherlands	36	1,131	37.11	20
2	Nazmi K	Vrije Universiteit Amsterdam	Netherlands	35	891	31.89	20
3	Nibbering PH	Leiden University	Netherlands	21	1,353	67.76	19
4	Valenti P	Sapienza University Rome	Italy	17	540	33.76	14
5	Yamauchi K	Morinaga Milk Industry Company, Ltd	Japan	19	1,107	60.11	17
6	Sanchez L	University of Zaragoza	Spain	18	353	21.61	11
7	Isobe N	Hiroshima University	Japan	18	69	7.44	8
8	Lonnerdal B	University of California Davis	United States	18	1,479	83.28	14
9	Vogel HJ	University of Calgary	Canada	18	3,012	170.50	15
10	Wakabayashi H	Morinaga Milk Industry Company, Ltd	Japan	18	1,170	67.28	16

^*^NP, total number of publications; NC, total number of citations; AC, average citations per item.

**Figure 4:**
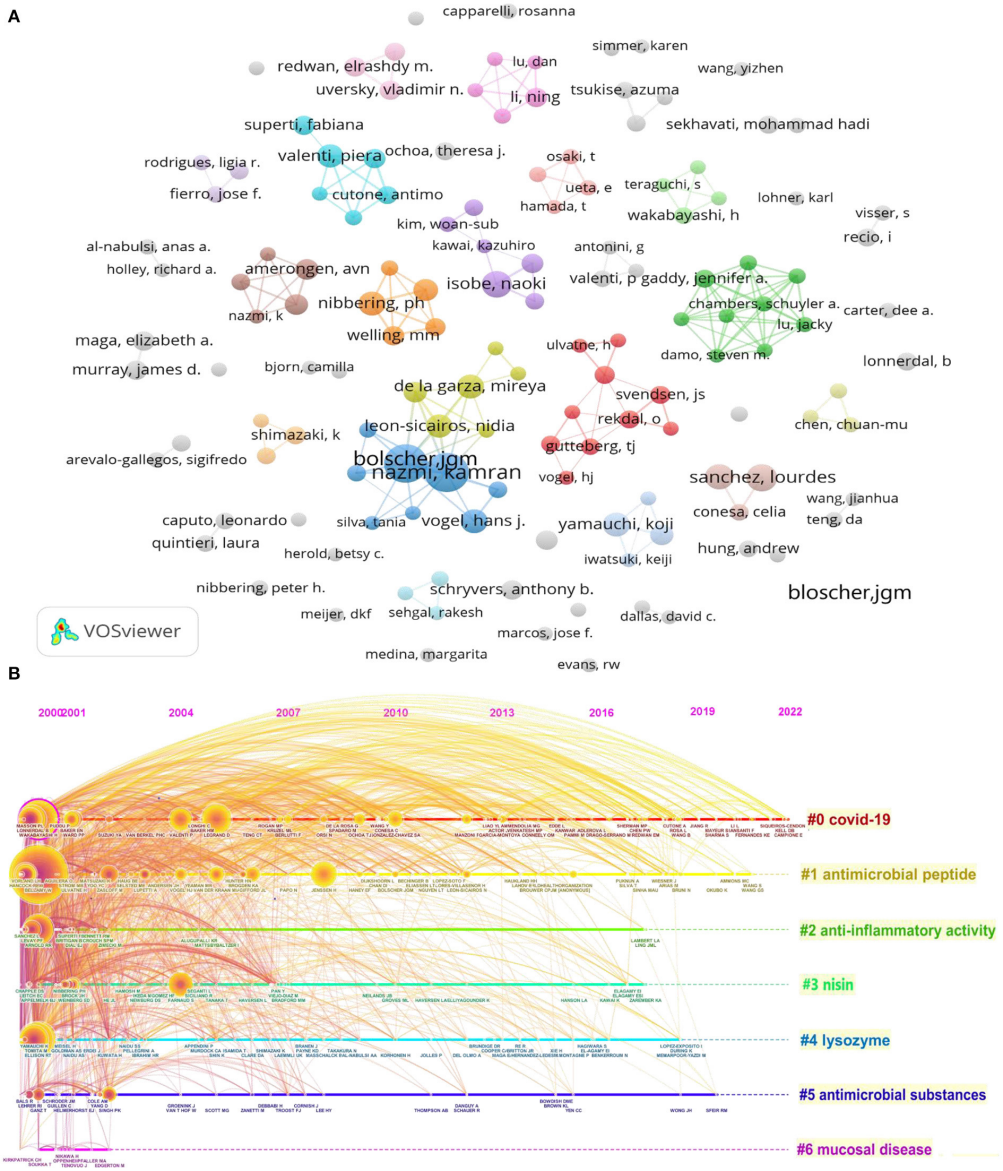
**(A)** Author coauthorship network visualization map. **(B)** Timeline view of Cocited authors cluster analysis in LF antibacterial research.

A network of coauthorship was produced using VOSviewer, as shown in Figure 4A. Bolscher JGM and Nazmi K are also the most cooperative with other authors. The data suggest that most authors publishing research on LF antibacterial research lack collaboration with other scholars.

Cocited authors are two or more authors who are simultaneously cited in one or more articles. These authors are related via co-occurrence. Among the 45,032 cocited authors, 160 had at least 50 citations. Figure 4B shows the timeline view of cocited author clusters, with their cluster labels on the right. Based on the cluster summary, coronavirus disease 2019 (COVID-19) caused by severe acute respiratory syndrome of coronavirus 2 (SARS-CoV-2) was the focus of recent attention. The popular themes that have emerged in recent years also include antimicrobial peptides, anti-inflammatory activity, nisin, lysozyme, antimicrobial substances, and the mucosal disease.

### Journals and cocited journals

Table 4 lists the top 10 most productive journals, their publishers, ISSN, NP, impact factor (IF), NC, and H-index. Most of the journals specialize in biomedicine and biochemistry. A total of six publishers are based in the United States, while two are based in England. *BioMetals* was the most prolific journal (NP = 46), and *Antimicrobial Agents and Chemotherapy* was the most influential journal (NC = 1,837, H-index= 26). The *International Journal of Molecular Sciences* had the highest IF (6.208), followed by the *Antimicrobial Agents and Chemotherapy* (5.938) and *Journal of Agricultural and Food Chemistry* (5.895).

**Table 4:** Top 10 most productive journals in the field of LF immunomodulatory of research.

**Rank**	**Journal**	**ISSN**	**Country**	NP*	IF-2021*	NC*	**H-index**
1	Biometals	0966-0844	Netherlands	46	3.378	1,706	24
2	Journal of Dairy Science	0022-0302	United States	41	4.225	902	19
3	Plos ONE	1932-6203	United States	39	3.752	1,047	20
4	Biochemistry and Cell Biology	0829-8211	Canada	33	3.730	1,687	18
5	Antimicrobial Agents and Chemotherapy	0066-4804	United States	31	5.938	1,837	26
6	Infection and Immunity	0019-9567	United States	23	3.609	1,673	22
7	International Dairy Journal	0958-6946	England	22	3.572	879	15
8	Journal of Agricultural and Food Chemistry	0021-8561	United States	18	5.895	502	12
9	International Journal of Molecular Sciences	1422-0067	Switzerland	15	6.208	281	7
10	Journal of Antimicrobial Chemotherapy	0305-7453	England	15	5.758	614	13

^*^NP, total number of publications; IF, impact factor; NC, total number of citations; AC, average citations per item.

**Figure 5:**
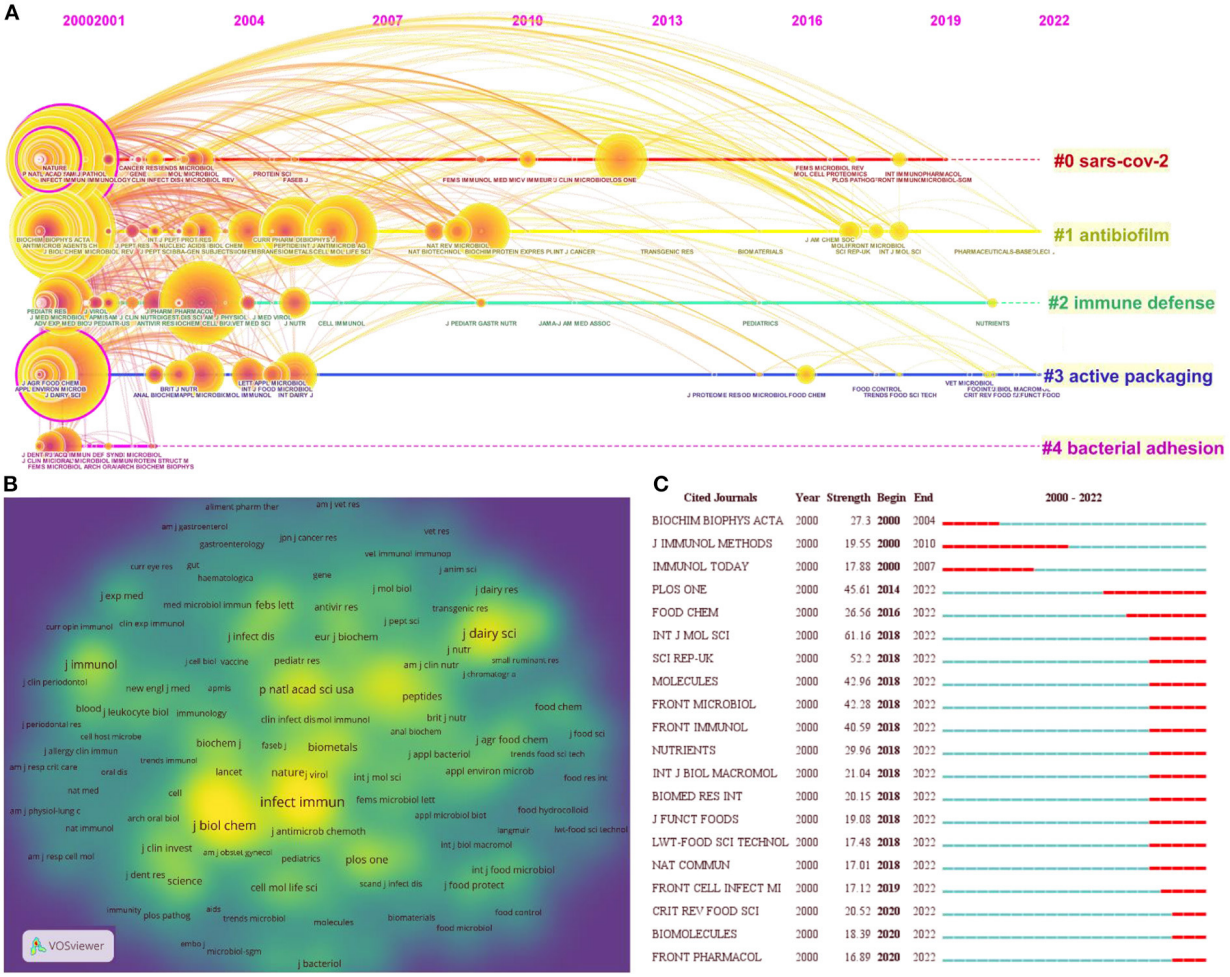
**(A)** Timeline view of journals cluster analysis in LF antibacterial research. **(B)** Density visualization for journals of cluster in cocited journals network map. **(C)** Top 20 cocited journals with strong occurrence bursts in LF antibacterial research.

Cocited journals are those in which two or more journals are cited concurrently by researchers. Figure 5A shows the timeline view of cocited journal clusters, with their cluster labels on the right. Based on the cluster summary, SARS-CoV-2, antibiofilm, immune defense, active packaging, and bacterial adhesion are current research hot spots in the LF antibacterial field. Figure 5B depicts the density visualization for the journals of clusters in cocited journals network map. Each journal in the density visualization has a color that indicates its density. Yellow means appearing more frequently, while green means appearing less frequently. *Infection and Immunity, Antimicrobial Agents and Chemotherapy, Journal of Biological Chemistry, Journal of Dairy Science*, and *Proceedings of the National Academy of Sciences of the United States of America* were the most five frequently and centrally cited journals. Figure 5C shows the top 20 cocited journals with strong occurrence burst in LF antibacterial research. *Critical Reviews in Food Science* and *Nutrition, Biomolecules and Frontiers in Pharmacology* were the journals with strongest occurrence burst in the last 3 years.

### Cocited references and reference burst

**Figure 6:**
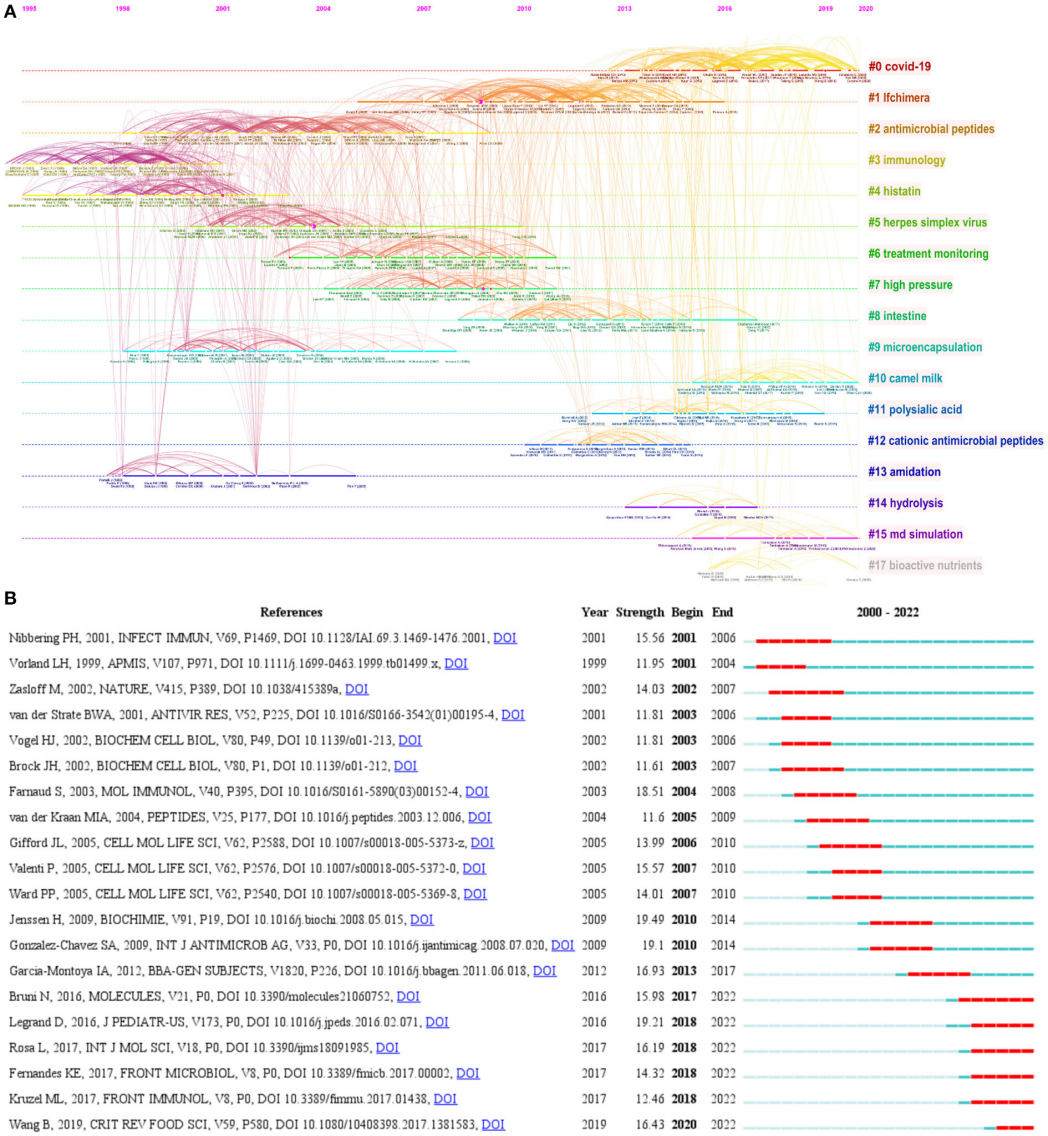
**(A)** Timeline view of cocited references cluster analysis in LF antibacterial research. **(B)** Top 20 cocited references with strong occurrence bursts in LF antibacterial research.

Among the 63,176 cocited references cited by the retrieved articles, 191 had at least 30 cocitations. Figure 6A shows a timeline view of the cocited reference clusters used to divide the articles into different clusters. Cluster 0 included the most references, which mainly focused on COVID-19, indicating that this topic was closely related to LF antimicrobial activity and needs further mining. The popular themes that emerged in the cocited references also included LFchimera, antimicrobial peptides, immunology, histatin, herpes simplex virus, treatment monitoring, high pressure, intestine, microencapsulation, camel milk, polysialic acid, cationic antimicrobial peptides, amidation, hydrolysis, MD simulation and bioactive nutrients. Figure 6B shows the top 20 cocited references with strong occurrence burst in LF antibacterial research. Wang B's article from 2019 about the structure, function, denaturation, and digestion of LF had the strongest occurrence burst (Wang et al., 2019).

### Citation analysis

**Figure 7:**
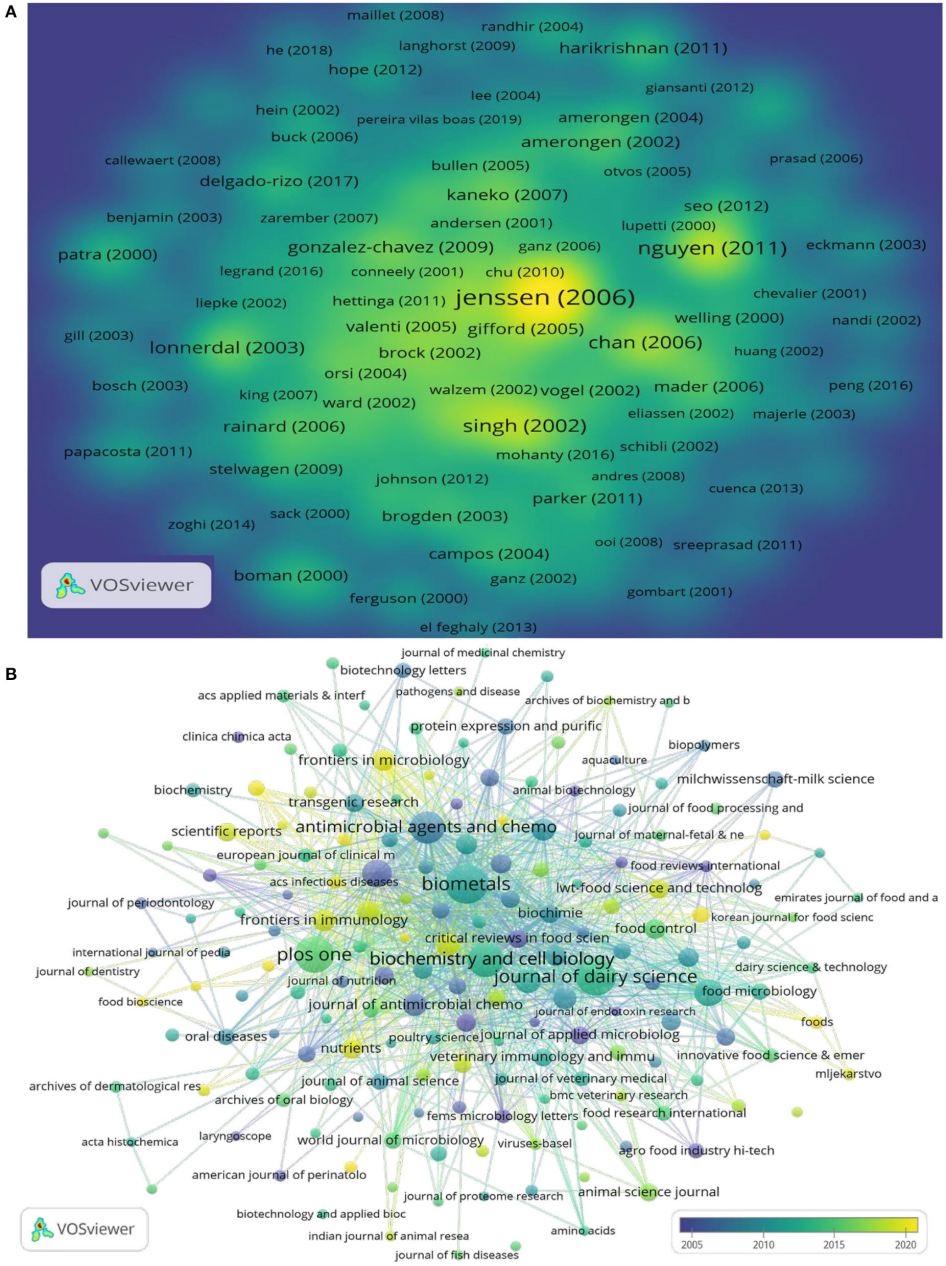
**(A)** Density visualization for citations authors cluster network map. **(B)** Citations journals co-occurrence analysis.

Of the 1,923 documents, 181 were cited more than 83 times. The VOSviewer density visualization map for citation author cluster network visualization is presented in Figure 7A. Yellow means appearing more frequently, while green means appearing less frequently. The highest total NC was found for the article written by Håvard Jenssen in 2006 (NC = 1,722) ranking first, followed by Leonard T. Nguyen's (NC = 952) and Pradeep K Singh's (NC = 735) articles.

Of the 770 document source journals, 176 journals were cited more than three times. Figure 7B shows the VOSviewer visualization map of cited journals in this field. The color of each circle indicates the average year when the journals appeared in articles, according to the color gradient in the lower right corner. The size of each circle indicates the frequency of occurrence of the journals. The distance between any two circles is indicative of their co-occurrence link, and the thickness of the connecting line indicates the strength of the link. *Antimicrobial Agents and Chemotherapy* was ranked first in terms of citations (NC = 1,866), followed by *BioMetals* (NC = 1,739), and *Biochemistry and Cell Biology* (NC = 1,720).

### Analysis of research hot spots

Keywords are the distillation of the research content of the literature and represent the theme of the article. When a keyword appears frequently, it reveals the distribution of scientific research topics and the hot spots and trends of research. Moreover, keyword analysis can also determine the time when a keyword with a change in frequency first appeared in a node, thereby defining the boundaries of the research field.

**Figure 8:**
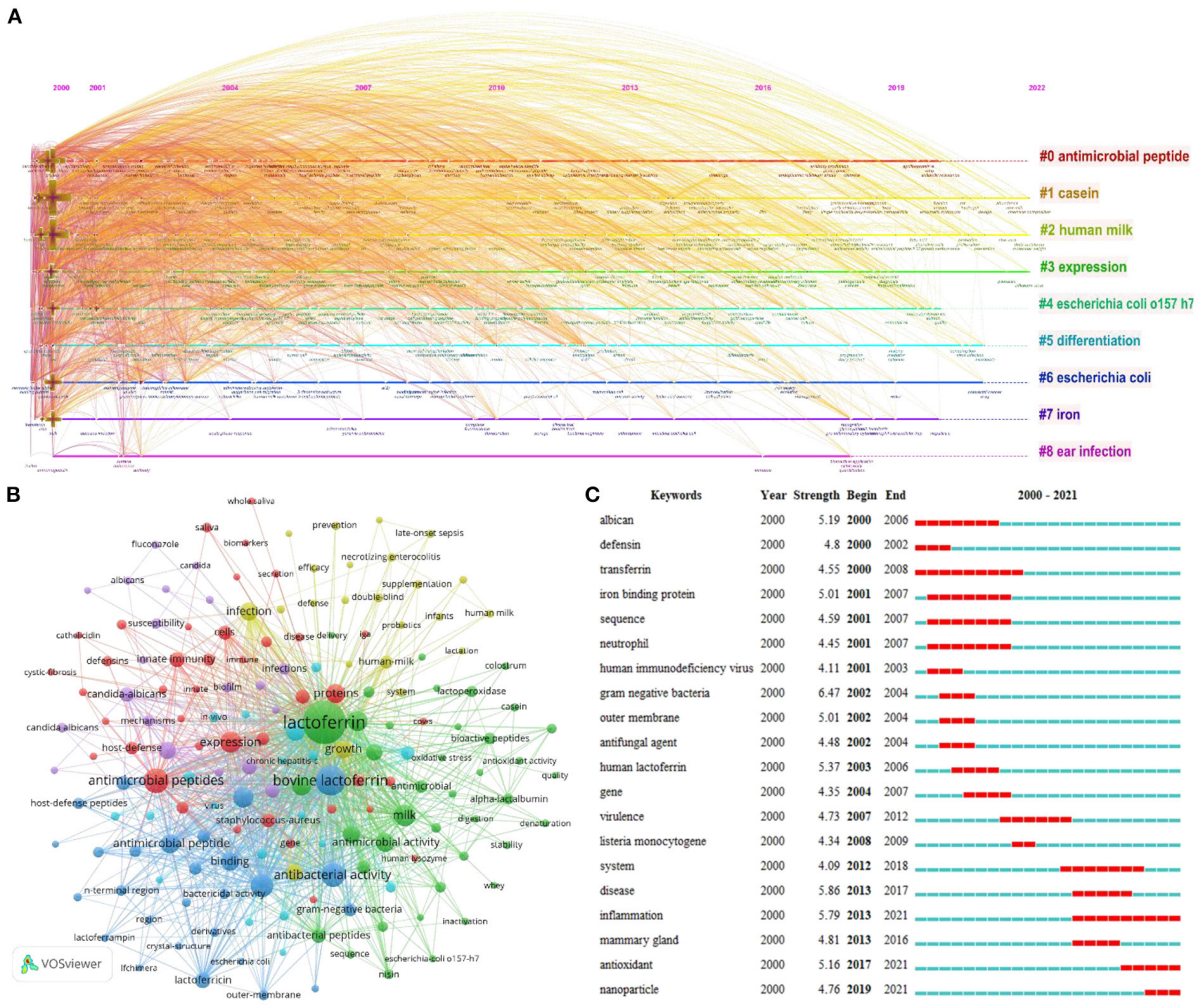
**(A)** Timeline view of keywords cluster analysis in LF antibacterial research. **(B)** Author keyword co-occurrence analysis. **(C)** Top 20 keywords with strong occurrence burst in LF antibacterial research.

Keywords extracted from the abstract of 1,923 articles were analyzed by CiteSpace and VOSviewer, respectively. CiteSpace was used to cluster keywords to identify hot research fields, as shown in Figure 8A. In total, nine hot research fields have been formed: “#0: antimicrobial peptide,” “#1: casein,” “#2: human milk,” “#3: expression,” “#4: Escherichia coli (O157:H7),” “#5: differentiation,” “#6: escherichian coli,” “#7: iron,” and “#8: early infection.” The smaller the number of clusters is, the more keywords are included, and each cluster is composed of multiple closely related words.

Between 2000 and early 2022, 8,292 keywords were used, in which the 170 keywords occurred more than 17 times, and these were divided into five clusters with different colors. The size of the node represents the frequency of occurrence. As illustrated in Figure 8B, cluster 1 (red) mainly focuses on the basic characteristics of LF and *in vitro* antibacterial activity mechanisms, cluster 2 (green) and cluster 3 (blue) reflected LF features and functions, and cluster 4 (yellow) and cluster 5 (purple) focused on LF sources.

Keyword emergence analysis can detect keywords with a high rate of change in frequency in the corresponding time node and determine the frontier of the research field. Figure 8C lists the keywords with the strongest occurrence bursts. As shown, the keywords with strong bursts from 2013 to 2022 included “disease,” “inflammation,” “mammary gland,” “antioxidant,” and “nanoparticle,” which represent emerging trends.

### Analysis of LF help defend against COVID-19

**Figure 9:**
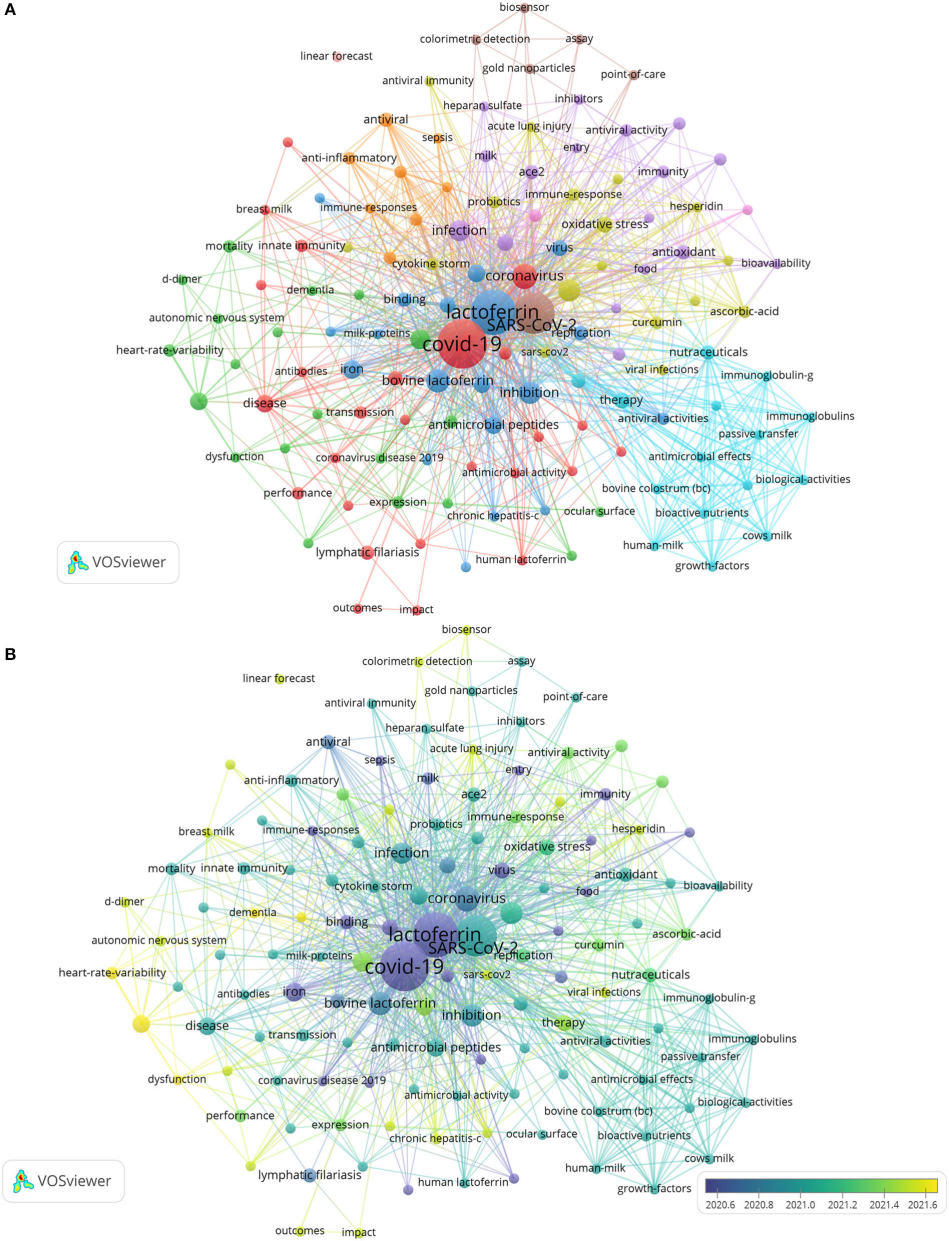
**(A)** Keyword co-occurrence analysis. **(B)** Keyword coauthorship overlay visualization map.

During the COVID-19 pandemic, the NP related to LF antibacterial activity increased sharply in 2020 (Figure 2A), which is also reflected in Figures 4B, 5A, 6A. From the 1,923 articles we searched, 131 articles on LF antibacterial activity related to COVID-19 were screened and analyzed to further understand the trend and hot spots about LF as potential preventative and adjunct treatment for COVID-19. As shown in Figure 9A, except for LF, COVID-19, and SARS-CoV-2, coronavirus, *in vitro* inhibition, infection, and iron and oxidative stress appeared most frequently. In addition, Figure 9A shows the involvement of LF in the pathogenesis and prevention of the virus from binding to the target cell surface to help defend against COVID-19. Moreover, the mechanism of antiviral treatment and immunomodulatory effects of LF therapy on COVID-19 and LF inhibiting COVID-19 progression were revealed (Figure 1A). In particular, there was considerable overlap between Figures 4A, 6A, suggesting that previous studies on LF antibacterial activity were similar to those on COVID-19. The keyword coauthorship overlay visualization map is shown in Figure 9B. The color of each circle indicates the average year when the keyword appeared in articles, according to the color gradient in the lower right corner. The size of each circle indicates the frequency of occurrence of the author keyword. The distance between any two circles is indicative of their co-occurrence link, and the thickness of the connecting line indicates the strength of the link. Therefore, we can expect to see future work dissecting these relationships, leading to many more important discoveries.

## Discussion

### General information

With the discovery of the antibacterial activities of LF, the potential value associated with drug application has attracted attention. The development of LF as a natural antibacterial has become an emerging research area. Having improved knowledge of the pharmacological properties of LF is crucial for future research. In this study, the antibacterial research status of LF from 2000 to early 2022 was analyzed for the first time, mainly using the quantity of publications, co-occurrence analysis, and keyword analysis.

In the past 22 years, trends in the NP about “antibacterial activities of LF” have significantly increased. This result indicates that the antibacterial activities of LF are attractive to researchers and will be a significant research direction (Figure 1A). According to the polynomial fitting curve, the annual NP trended generally upward and increased in the latter half of the period, particularly after 2019 (Figure 1B). The COVID-19 outbreak indirectly led to an annual increase in the NP, which maybe the main reason for this result. From 2000 to early 2022, the most productive country publishing articles on the antibacterial activities of LF was the United States. In China, research on the antibacterial activities of LF was performed slightly later but developed quickly, making it second in the NP on antibacterial activities of LF. However, compared to the those of the aforementioned countries, its NC and H-index remain low. In this case, Chinese scholars and institutions need to focus more to improve their research and to make efforts to improve research quality.

Analysis of collaboration networks revealed collaborations between different countries. The United States has the most cooperation with other countries. This partially explains why the United States has developed rapidly in this field over the past 22 years. Likewise, most high-income countries such as Italy, Germany, England, the Netherlands, and Poland are also in the middle of the cluster map, indicating that these countries continue to commit to and invest in this field. Therefore, China should pay more attention to effective cooperation among different countries and institutions to increase the level and quality of research related to the antibacterial activity of LF.

From the top 10 productive institutions, four institutions and three authors were located in the Netherlands, which suggested that the Netherlands has been one of the pioneers in researching the antibacterial effects of LF. Håvard Jenssen was the most cited author publishing articles on the antibacterial activities of LF and focused mainly on the relationship between the antimicrobial peptide structure and function, as well as the mechanism of action (Jenssen et al., 2006). In addition, other frequently cited works illustrated different LF antimicrobial mechanisms (Singh et al., 2002; Nguyen et al., 2011). Yamauchi K and Wakabayashi H (Morinaga Milk Industry Company, Ltd) are among the top 10 active authors from dairy enterprise. Notably, Morinaga Milk is the first commercial organization to advocate the addition of LF to the infant formula in 1986 (Oda et al., 2014). Currently, bovine LF is used as an additive ingredient in various foods, such as infant formula, yogurt, skim milk, and beverages. The role of LF in human milk has much clinical evidence, while most of the antibacterial research studies on bovine LF are premised on *in vitro* experiments. Whether LF added to formula milk or directly supplemented can prevention or treatment of neonatal infections is still controversial in clinical studies (Demmelmair et al., 2017; Pammi and Suresh, 2020). After all, adding LF to milk powder can improve children's immunity, which so far has not been officially endorsed by authorities such as the FDA. Currently, there is no complete systematic risk assessment of the safety, bioavailability, or functional differences of LF that may have adverse health effects in infants, and the interaction of LF with other ingredients in milk powder and products research on stability factors. These may be application challenges that researchers need to solve in future.

Among the top 10 journals publishing on the antibacterial activities of LF, only four had an IF >5. The number of articles published in these journals accounted for 14.7% of the total publications included in this study. This result indicates that publishing research on LF antibacterial in high-quality journals is a challenge. To date, the most relevant subjects of the antibacterial activities of LF include microbiology, food science, molecular biology, structural biology, and nutrition, which may continue to be the main subjects of focus in future studies.

The 20 articles with a high NC were published in journals with a high IF, indicating that these journals have published a greater number of potential breakthroughs in this field. As a result, researchers interested in this field should pay more attention to the recent publications in these journals.

### Emerging topics

Hot spot analysis helps explore research frontiers and trends. From the analysis of the keyword map (Figure 7), it is concluded that the current research hot spot in the field is gradually shifting from the research on function, structure, and mechanism to application. Studies have shown that the various active functions of LF do not exist in isolation, and they exist in synergistic or causal relationship between them. LF and LF-derived peptides probably kill bacteria in different ways: high iron affinity, which limits iron availability to microorganisms (Nairz et al., 2010); specifically interact with receptors on target cells to block the transport of nutrients, thereby inhibiting bacterial synthesis and metabolism (Appelmelk et al., 1994); and exert antibacterial activity by inhibiting enzyme activity (Brock, 2012). The latest research showed bovine LF against the potato common scab pathogen Streptomyces scabiei due to its short active peptides, and LF-derived bioactive peptides might be an effective and alternative antimicrobial substances (Nakamura et al., 2022). In addition, LF can also be used as emulsifiers and texture modifiers to be an ideal nanocarrier for some hydrophobic therapeutics (Sabra and Agwa, 2020). Fabrication of LF-based nanocarriers can specifically deliver drugs to the brain across the blood–brain barrier and extensively enhance the therapeutic efficacy of the encapsulated active molecules (Zhao and Yang, 2018; Agwa and Sabra, 2021). Therefore, LF may also be a promising molecule for cancer therapy and nanomedicine (Duarte et al., 2022).

Due to the SARS-CoV-2 outbreak leading the COVID-19 pandemic, the NP of this field after 2019 reached the highest level, which was verified by cluster 0 in Figures 4B, 5A, 6A. As a multifunctional protein with a broad-spectrum of antimicrobial activities, the molecular docking screening results indicate that LF can be used as a potential drug for the treatment of COVID-19 and also confirmed that a reasonable dose of LF exhibits anti-viral properties in SARS-CoV-2-infected cells*;* LF combined with diphenhydramine can reduce the replication of the SARS-CoV-2 by 99% and can also shorten the recovery time of the new crown infection (Mirabelli et al., 2021; Ostrov et al., 2021). LF inhibits SARS-CoV-2 infection in cell models with multiple modes of action, including blocking viral attachment to cellular heparan sulfate and enhancing interferon responses (Hu et al., 2021). Furthermore, *in vitro* tests show that the bovine LF product is effective against variant SARS-CoV-2 strains (including SARS-CoV-2 variants WA1, B.1.1.7, B.1.351, P.1, and B.1.617.2) (Wotring et al., 2022). The antiviral effect of LF is mediated by preventing the virus binding to the target cell surface, which would be particularly effective during the early amplification phase of the virus in the salivary glands, throat, and upper respiratory tract (Wakabayashi et al., 2014). According to Figure 5A, the “cytokine storm” is one of the main pathogenesis mechanisms of SARS-CoV-2 virus-induced COVID-19. Therefore, inhibiting the cytokine storm may be a prophylactic and therapeutic approach toward combating COVID-19 infection (Zimecki et al., 2021). In the study of pulmonary acute respiratory distress syndrome (ARDS) in granulomatous inflammation model (Mrityunjaya et al., 2020), it was found that LF can reduce or eliminate cytokine excess and pulmonary pathological features caused by *Mycobacterium tuberculosis* (Welsh et al., 2011; Hwang et al., 2017). In addition, LF was also able to diminish hyperacute immunopathology developed after the administration of isolated surface mycolic acid components (Nguyen et al., 2021, 2022). LF significantly induced the expression of the antiviral immune response genes and partially inhibited SARS-CoV-2 infection and replication in Caco-2 intestinal epithelial cells to protect against SARS-CoV-2 infection *in vitro* (Salaris et al., 2021). After LF treatment, pro-inflammatory factors (TNF-α, IL-1β, and IL-6) significantly decreased, while anti-inflammatory factors (IL-10 and TGF-β) were maintained or even enhanced (Wang et al., 2021). Overall, these recent research hot spots may make LF an exciting clinical candidate for the treatment or prevention of SARS-CoV-2 in future. Of course, similar to development of it into an antibacterial drug, there are still numerous challenges from clinical application. It is also necessary to overcome the bioavailability, active substance basis or function differences, and safety and clinical trials. Undoubtedly, this will require researchers to demonstrate their studies in future.

### Limitations

This study is based on bibliometric and bibliographic visualization analyses of the literature, which may aid researchers in gaining a better understanding of the development trend and academic frontiers of the field. In addition, this study employs the NC as an indicator, which may help scholars comprehend significant nodes in the trend in this field. Nonetheless, this study has some limitations. First, only English language articles and reviews from SCI Expanded-indexed journals were included. Second, some details may be omitted due to VOSviewer inability to analyze the full text of publications. Last, some newly published excellent articles with a low NC may be excluded due to lag. We hope that future studies will look at more databases and obtain a complete picture of the antibacterial properties of LF worldwide.

## Conclusion

This study used bibliometric analysis to summarize the articles on the antibacterial effect of LF. It sheds light on the evolution of publications and their citations on the antibacterial effect of LF over the last 22 years. Generally speaking, as a broad-spectrum antibacterial peptide with great potential for research and drug development, LF requires further attention in frontier studies on nanomedicine, druggability, clinical validation, and basic research, and safety and efficacy also need attention as well.

## Author contributions

YLX: conceptualization, methodology, and writing—original draft. XYL and JLH: formal analysis, data curation, validation, and writing—review and editing. YJW: supervision and project administration. WPZ: funding acquisition. All authors have read and agreed to the published version of the manuscript.

## Conflict of interest

The authors declare that the research was conducted in the absence of any commercial or financial relationships that could be construed as a potential conflict of interest.

## Publisher's note

All claims expressed in this article are solely those of the authors and do not necessarily represent those of their affiliated organizations, or those of the publisher, the editors and the reviewers. Any product that may be evaluated in this article, or claim that may be made by its manufacturer, is not guaranteed or endorsed by the publisher.
